# Evaluation of the Biophysical Signs of Healing in the Protocolized Use of the High Capillarity Dressing: A Pilot Study

**DOI:** 10.1111/iwj.70332

**Published:** 2025-04-16

**Authors:** Miguel Mellado Sanz, Carmen Alba Moratilla, Merecedes Martínez Delgado, Manuel Gerónimo Pardo, Roi Painceira‐Villar, Bibiana Trevissón‐Redondo

**Affiliations:** ^1^ Heridea Clinic Palencia Spain; ^2^ eSalúdate Valencia Spain; ^3^ University of Valladolid Valladolid Spain; ^4^ Department of Anesthesiology Integrated Health Management of Albacete Albacete Spain; ^5^ Department of Nursing and Physiotherapy, Faculty of Health Sciences University of León León Spain

**Keywords:** chronic wound healing, high‐capillarity dressing, hyperspectral imaging

## Abstract

The aim of this study was to evaluate the effects of a high‐capillarity dressing on chronic wound healing, using hyperspectral imaging to assess biophysical parameters. This quasi‐experimental pilot study involved eight adults with chronic ulcers treated with the high‐capillarity dressing for 4 weeks. Primary outcomes included pH, temperature, and oxygen saturation, measured using hyperspectral imaging, along with wound area assessed with ImageJ. Secondary outcomes included quality of life, evaluated using the EQ‐5D‐5L and Wound QoL‐17 scales. Data were collected at baseline, 7, 14, and 21 days, and analysed using SPSS. Results showed a significant reduction in lesion size (*p* < 0.05) and pain (*p* < 0.001) following treatment. Biophysical assessments revealed a significant decrease in pH (*p* = 0.004), but no significant changes were observed in other parameters (oxygen saturation, NIR, TWI, TLI). A significant correlation was found between oxygen saturation and pH (*p* < 0.005). The results suggest that the high‐capillarity dressing improves chronic wound healing by reducing lesion size, promoting pH acidification, and improving superficial oxygenation. Additionally, the dressing controlled edema and eliminated infection signs without antibiotics, suggesting its potential in optimising the wound healing environment. These findings highlight the need for further research into the clinical application of this dressing in chronic wound management.

**Summary**

- The aim of this study was to evaluate the impact of a high‐capillarity dressing on chronic wound healing using hyperspectral imaging.
- An eight‐participant pilot study was conducted with chronic ulcer patients treated for four weeks. pH, temperature, oxygen saturation, and wound area were measured.
- Results showed significant reductions in wound size and pain. pH decreased, and a correlation was found between pH and oxygen saturation. The dressing also controlled edema and eliminated infection signs without antibiotics.
- In conclusion, the high‐capillarity dressing improves healing, showing potential for chronic wound management.

## 1 Introduction

The assessment of wound healing is generally based on subjective criteria provided by the observer, such as the type of tissue present in the wound bed, the characteristics of the wound edges, the surrounding skin, and the quantity and type of exudate [1]. Eliminating this subjectivity and the associated observer error would significantly improve the clinical decision‐making process for proper wound management. The development of objective tools for assessing healing would enable a more efficient and standardised therapeutic approach [2].

The objective study of the phases of the healing process, the types of cells involved, as well as the biophysical factors affecting its progression has become highly relevant in current research. Among these factors, pH, temperature, oxygen saturation, and various biophysical indices such as the Tissue Perfusion Index (NIR), Subcutaneous Water Index (TWI), and Tissue Lipid Index (TLI) stand out. These metrics provide a more accurate view of the physiological variations occurring during healing, allowing for improvements in diagnostic and therapeutic methods [3].

This quantitative approach is essential to improve the accuracy of clinical evaluation and decision‐making related to wound treatment, especially in patients with chronic diseases such as diabetes [4].

The use of instruments capable of measuring biophysical parameters such as pH, temperature, oxygen saturation, tissue perfusion, subcutaneous water, and tissue lipid indices offers a crucial advantage for diagnosis and clinical decision‐making. These instruments provide clinicians with additional information about the state of healing, enabling more precise and effective intervention. Among the most promising technologies is the hyperspectral light lamp, which offers several advantages over traditional methods like thermography or indocyanine green angiography. Its main advantage lies in its ability to measure multiple parameters in a single exposure and in a non‐invasive manner, reducing both cost and patient discomfort [5, 6].

However, a limiting factor of these advanced tools is their high cost, restricting accessibility for many clinicians, particularly in resource‐limited areas [6].

This approach is supported by studies highlighting the utility of hyperspectral spectroscopy in assessing tissue perfusion and other key parameters in wound healing, compared to invasive and costly techniques like angiography [7].

The pH of intact skin has been widely studied, with values typically ranging from 4.5 to 6, maintaining a slightly acidic environment crucial for its protective barrier function. However, when a lesion breaks the epidermis, the pH increases to more alkaline levels, around 7–7.5. This initial change is a natural response of the body to tissue damage [6, 7].

In chronic wounds, it has been observed that the pH remains elevated during the early stages of healing, suggesting an alkaline environment. As the healing process progresses, the pH tends to neutralise and then return to acidic levels. Persistent alkaline pH is often associated with wound chronicity and poor healing. This parameter is highly valuable diagnostically, as a sustained alkaline pH could indicate a chronic state that requires specific interventions to promote healing [6, 7].

pH also plays a crucial role in oxygen release, as a decrease of 0.9 units in pH can increase oxygen saturation up to five times. This process promotes angiogenesis, a key component in the formation of new blood vessels, and modulates the activity of proteases and other pro‐inflammatory mediators involved in the various stages of wound healing. These combined effects underscore the importance of maintaining a controlled acidic environment to optimise tissue repair [8].

It has been shown that most bacterial strains have an optimal pH range for growth between 7.0 and 7.5. However, bacteria can proliferate across a much wider pH range, from 4.5 to 9. This means that the acidic pH of intact skin is not sufficient to completely prevent bacterial growth, and this vulnerability increases when the skin barrier is compromised [6, 7]. Various pathological skin conditions cause pH alterations, which may increase susceptibility to infections. For example, in seborrheic dermatitis, affected areas have a higher pH compared to the rest of the skin, creating a favourable environment for pathogen growth. Similarly, diabetic patients often show a tendency toward elevated pH in their skin, promoting the growth of *Candida albicans* , a common fungus that can complicate the management of skin infections in these patients [9]. Monitoring pH during the healing process can provide valuable information about wound progression and enable more accurate treatment decisions. Maintaining pH control helps identify the wound's status, as elevated pH levels are typically associated with poor healing or chronicity, while a tendency toward acidification indicates a more favourable healing process [10, 11].

The hyperspectral lamp is designed to measure the chromophores of haemoglobin and its derivatives: oxyhemoglobin, deoxyhemoglobin, and tissue water. This allows the creation of an oxygenation map, both cutaneous and subcutaneous, through StO_2_ at 0.8 mm from the surface and the Tissue Perfusion Index (NIR) at 2.6 mm [8, 12].

Oxygen plays a critical role in the wound healing process. In fact, if tissues do not receive adequate oxygen perfusion, healing may be compromised [8]. As a result of this observation, various therapies have been developed, such as transdermal oxygen application, aimed at improving tissue oxygenation and facilitating healing. Monitoring oxygen saturation (StO_2_) and the perfusion index provides a more detailed view of how tissues evolve, offering a more objective assessment of treatment effectiveness, including dressings used to promote healing (NIR).

Temperature is a key biophysical marker that can provide essential information on wound progression. The optimal temperature for promoting healing ranges between 33°C and 42°C. When wounds fall outside this range, healing is likely to be delayed. Specifically, in wounds, the temperature typically ranges between 24°C and 26°C due to the loss of the tissue barrier, resulting in greater evaporation and cooling of the affected area [13, 14, 15].

The temperature of the perilesional skin offers additional insight into the state of the lesion. A difference of more than 2°C between the wound bed and the surrounding skin temperature may indicate infection or inflammation. Temperature can also influence pH measurement, as it affects ion activity. An increase in temperature raises the solubility of salts, acids, and bases, leading to a higher concentration of H+ and OH− ions [13, 14].

Current research in wound healing is advancing toward a deep understanding of the biological and physical markers involved in the process, as well as the development of innovative products like advanced dressings and bioactive substances. Rigorous monitoring of these parameters, in relation to the use of a specific healing product, will not only allow a more accurate evaluation of its effectiveness but also redefine clinical standards for the treatment of complex wounds. In our study, we analyse how key healing markers are modified by the application of a high capillarity dressing capable of generating an exudate absorption pressure of −68 mmHg. This advancement promises a significant impact on improving healing times and reducing infectious complications, positioning such dressings as a revolutionary option in the management of difficult wounds.

Therefore, the general objective of the study was to evaluate the effectiveness of a high‐capillarity dressing in chronic wound healing through the analysis of biophysical healing parameters.

## 2 Materials and Methods

### 2.1 Study Design

Pilot descriptive, quasi‐experimental case series study.

### 2.2 Ethics Statement

The study was approved by the Research Ethics Committee of the University YYYYYYYY under internal registration number ETICAXXXXXXXXX. Written informed consent was obtained from all volunteers after explaining the study procedures. Patients were assigned an alphanumeric code to maintain anonymity, in compliance with Organic Law 3/2018 of December 5 on data protection and digital rights. This research complies with all regulations related to human experimentation and the considerations outlined in the Helsinki Declaration [16].

### 2.3 Sample

For this pilot study, a consecutive convenience sampling method was applied to an adult population. Eight patients with ulcers classified as chronic from various aetiologies were selected in a wound care clinic, with their treating healthcare professional overseeing the treatment. These patients were treated between May 2024 and September 2024 and met the inclusion criteria: patients with chronic ulcers that had not responded to treatment for at least 4 weeks prior to inclusion, or post‐amputation wounds with or without exposed tendon/ligament/bone, who had signed informed consent. Patients with moderate to severe ischaemia (ABI < 0.6 or non‐palpable peripheral pulse), those who had participated in other wound‐related trials within the last 30 days, or with suspected malignancy were excluded.

### 2.4 Sociodemographic and Descriptive Data

Sociodemographic and descriptive variables collected included age, gender, medical history, chronic treatment history, lifestyle habits, ankle‐brachial index, and pain levels.

### 2.5 Primary Outcome Measures

Primary variables included the number of lesions, location, aetiology, time of evolution, start and end dates of treatment, reasons for discontinuation, signs of superficial and deep infection, wound culture and results, allergic reactions, pain, wound area in cm^2^, pH, temperature, wound oxygenation (StO2), NIR, TWI, TLI.

Clinical signs of infection were assessed using the NERDS and STONES scales for chronic wound bacterial load and infection [17]. The NERDS scale, which considers five clinical signs (healing, exudate, friable tissue, debris, and odour), was applied to identify local infections, while the STONES scale, with six criteria (size increase, temperature, bone exposure, new areas of breakdown, exudate, and erythema/edema/odour), was used to assess invasive infections.

For the determination of the wound area, the “ImageJ” software (National Institute of Health, USA) was used, an open‐source image analysis tool validated in clinical studies for its accuracy and versatility [18]. ImageJ allows for objective and reproducible area measurements, reducing the subjectivity inherent in visual assessments. In this study, the ulcer images were captured under standardised conditions and then processed in ImageJ, where the contour of each lesion was traced, and the area was calculated in square centimetres.

Finally, for the quantification of temperature, tissue oxygenation, NIR, TWI, and TLI, the TIVITA hyperspectral imaging system was used, allowing for a precise characterisation of tissue status [19].

### 2.6 Secondary Outcome Measures

For the evaluation of quality of life, the EQ‐5D‐5L scale [20] and the Wound QoL‐17 scale [21] were used.

The EQ‐5D‐5L scale is a standardised tool for assessing health‐related quality of life, developed by the EuroQol Group. This scale analyses five key dimensions: mobility, self‐care, daily activities, pain/discomfort, and anxiety/depression, each with five levels of severity. It also includes a visual analog scale (VAS) from 0 to 100, where patients indicate their overall health perception. This scale allows for a detailed analysis of the impact of chronic ulcers and their treatments on the patient's quality of life.

Lastly, the Wound‐QoL‐17 was used, which is a specific scale for evaluating quality of life in patients with chronic wounds. Composed of 17 items, this scale is designed to capture the physical, psychological, and social impact of living with a chronic wound. The items are grouped into three dimensions: physical symptoms (pain, discomfort), daily limitations (restrictions on activities and mobility), and psychological impact (worry and frustration). Patients score each item on a Likert scale, which allows for quantifying the degree of impact in each area and calculating an overall quality of life score related to the wound.

### 2.7 Statistics

Data were processed using the SPSS statistical software (version SPSS 29.0.2.0; IBM; Armonk‐NY; IBM Corp., USA). Descriptive analysis for quantitative variables was performed using central tendency measures such as mean, median, and mode, as well as their standard deviation, range, and quartiles. For qualitative variables, percentages and frequencies were used. For studying correlations between variables, Spearman's Rho and the *t*‐Student test were used with a 95% confidence level. Statistically significant values were set at *p* < 0.05.

### 2.8 Procedure

An observation period of 4 weeks was established. Controls and data collection were carried out at the beginning, on days 7, 14, and 21, regardless of the frequency of dressing changes.

A data collection notebook was used for the different variables.

**FIGURE 1:**
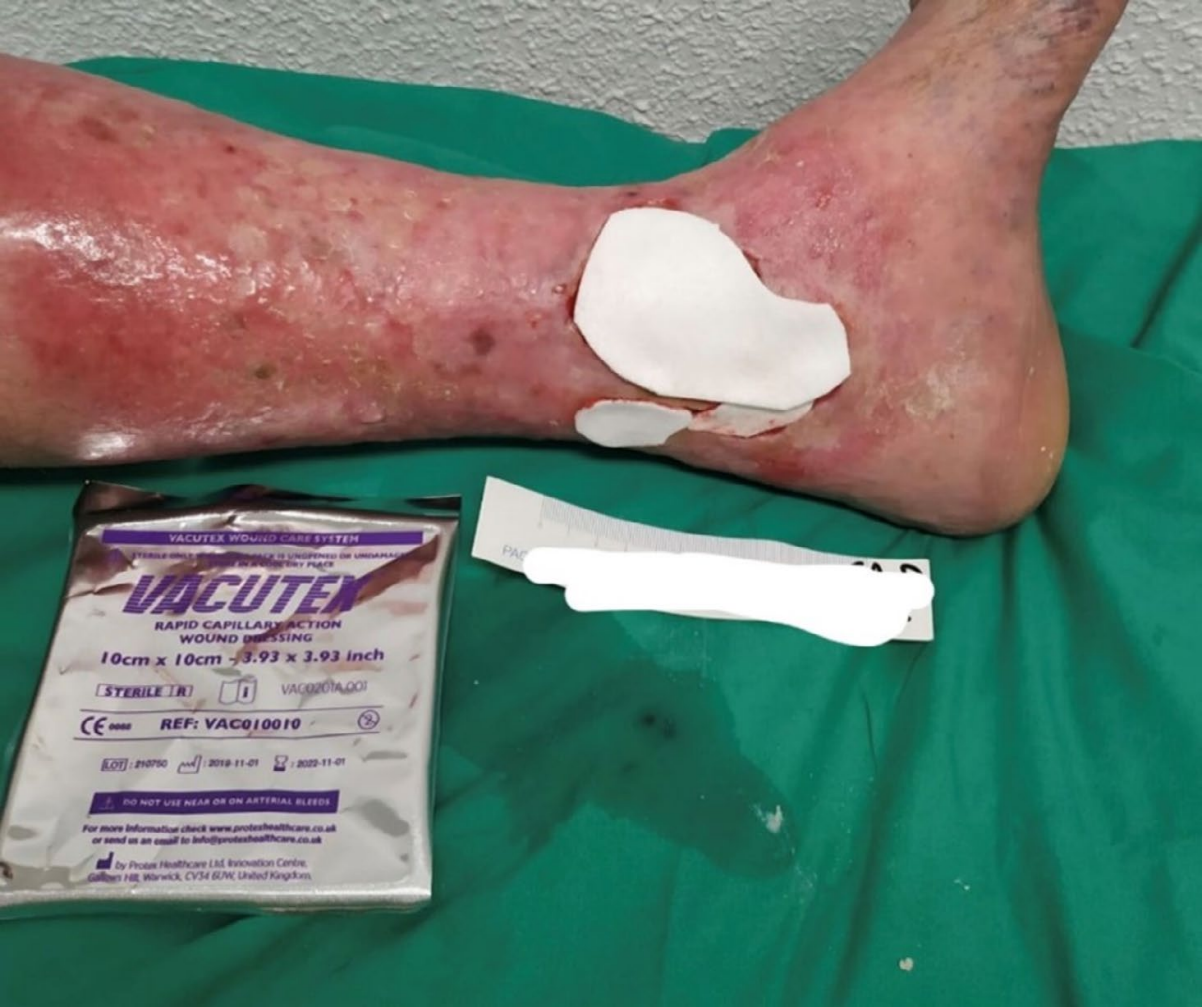
High‐capillarity dressing used and its application in one of the study subjects. Image of the dressing used in the study, showing its application on one of the participating subjects. The dressing was applied according to the established protocol to evaluate its effectiveness and safety in the subjects included in the study. *Source:* Own data.

The dressing procedure used is part of the routine practice in the wound care clinic participating in the study. The frequency of dressing changes was determined by the investigator as necessary for each patient. The dressings were applied as indicated without using other techniques or products that could interfere with the wound healing process: First, the wound was cleaned with saline solution, followed by taking images to later measure the wound area. Then, the previously described variables were recorded, and the area to be treated was cleaned with antiseptic wipes, followed by the application of a high‐capillarity dressing (VACUTEX) and, finally, a secondary dressing (calcium alginate fibre). Figure 1 shows the application of the selected high‐capillarity dressing.

## 3 Results

Eight patients were selected from a private wound care clinic between February 2022 and May 2023, consisting of 3 males and 5 females, all over the age of 50. All of them had at least one wound and one concomitant chronic disease. Regarding harmful lifestyle habits, the most frequent were tobacco, alcohol, and/or drug use, prolonged standing, and sedentary behaviour. All patients had at least one wound, with a minimum duration of 3 months. The aetiology of the lesions was venous, and one of them had an ischaemic component. The locations were the leg and foot. A vascular examination was performed using the ankle‐brachial index (ABI); one patient had an ABI > 1.3, above the normal range, and in two cases, the ABI was in the range of (0.8–0.6), considered moderate Peripheral Arterial Disease (PAD), while the rest had an ABI within the normal range.

Regarding clinical signs of superficial infection (NERDS) and deep infection (STONES), four patients had at least two signs of deep infection. Three microbiological cultures were performed, all three yielding positive results, with *Staphylococcus aureus* as the causative germ in all three cases, and one case also had *Pseudomonas aeruginosa* . In subsequent follow‐ups, none of the patients showed clinical signs of superficial or deep infection.

One patient voluntarily abandoned the treatment during the first week, four cases showed complete epithelialisation during the observation period, and in the remaining three cases, there was good progress, although full epithelialisation had not occurred by the end of the observation period.

**FIGURE 2:**
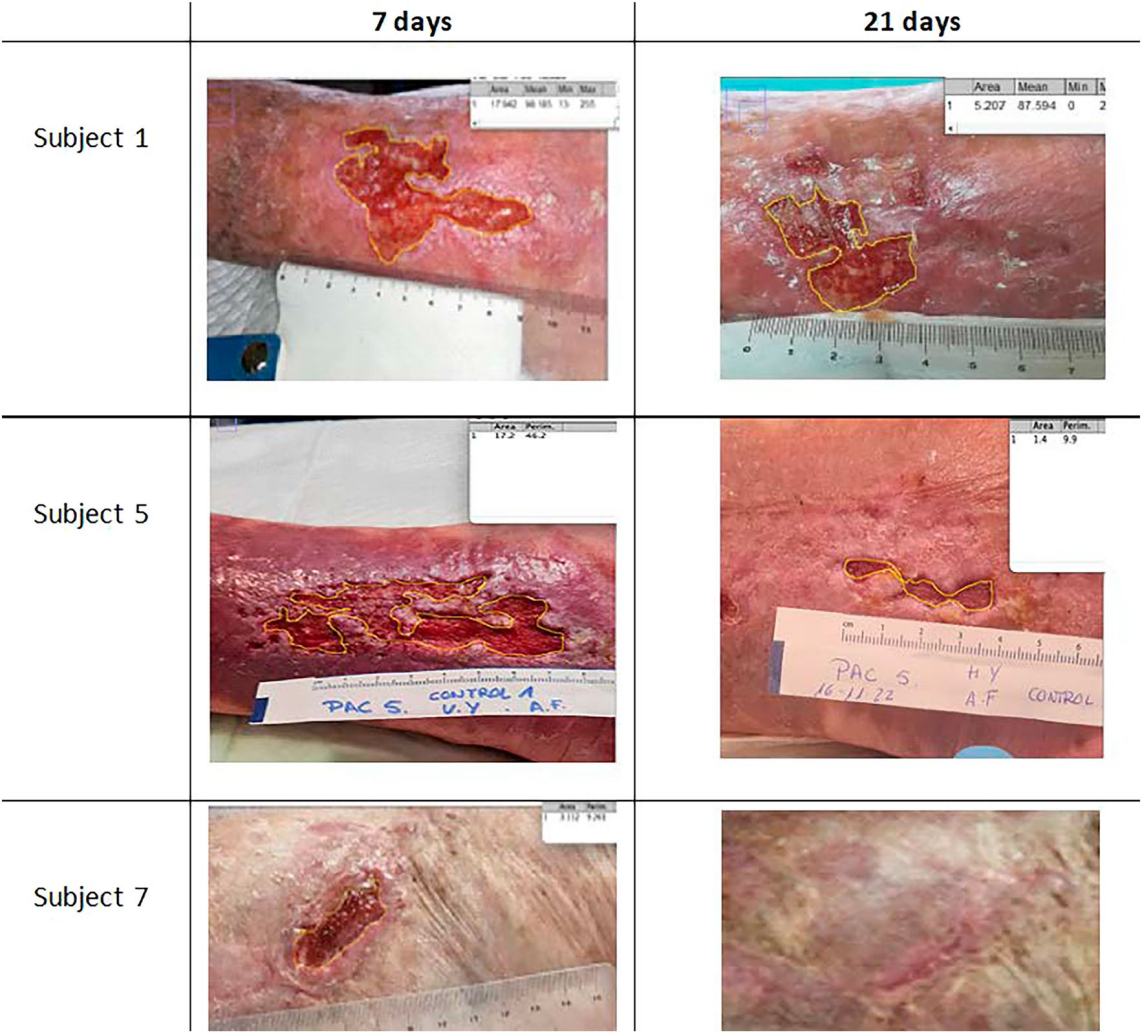
Series of representative wound photographs illustrating the progression of healing across multiple cases. Healing progression over time: Comparison of subjects at 7 and 21 days. The figure displays the wound length and area measurements at 7 and 21 days. *Source:* Own data.

Figure 2 shows a series of representative wound photographs, demonstrating the progression of healing through the reduction in wound size across several cases. These images illustrate how, over time, the lesions gradually decrease in size, reflecting the evolution of the healing process in the different cases analysed.

The healing progression is shown in Table 1, with a statistically significant reduction in the size of the lesions. The mean difference was statistically significant, with a significance of (*p* < 0.05).

**TABLE 1:** Evolution of wound size, lesion temperature, perilesional temperature, and SatO_2_.

	1st control *n* = 8	2nd control *n* = 7	3rd control *n* = 7	4th control *n* = 7
Wound size (cm^2^)	12.813 ± 9.144	10.057 ± 7.087	6.785 ± 8.446	2.885 ± 4.424
Lesión temperature (°C)	31.26 ± 1.455	31.21 ± 1.764	30.44 ± 1.355	30.70 ± 0.721
SatO_2_	57.0 ± 16.28	66.5 ± 10.89	65.0 ± 8.48	61.3 ± 15.85
Perilesional temperature (°C)	31.10 ± 1.506	31.48 ± 0.885	31.128 ± 0.930	30.866 ± 1.076

*Note:* Results of the controls performed at different time points (1st to 4th control) show the mean ± standard deviation for wound size, lesion temperature, SatO_2_, and perilesional temperature. The table indicates changes over time in the wound area, temperature of the lesion, oxygen saturation, and the temperature surrounding the lesion.

Abbreviation: SatO_2_, Oxygen saturation.

On the other hand, pain showed a significant decrease throughout the observation period with statistical significance (*p* < 0.001). Regarding pH, a decrease of 1.00 ± 0.477 was observed, with statistical significance (*p* = 0.004). SatO_2_ increased by 5.5 ± 6.745 from the first to the last observation, though without statistical significance. The NIR index for subcutaneous perfusion showed a mean difference of −10.00 ± 8.221, without statistical significance. The TWI index for subcutaneous water (as shown in Image 1) decreased with a mean difference between the first and fourth observation of 10.66 ± 11.792, and the TLI index decreased with a mean difference of 8.50 ± 15.782, but neither showed statistical significance. These results are summarised in Table 2.

**TABLE 2:** Evolution of biophysical signs.

	1st control *n* = 8	2nd control *n* = 7	3rd control *n* = 7	4th control *n* = 7
Pain	7.25 ± 0.707	4.29 ± 0.951	2.57 ± 1813	0.83 ± 0.753
pH	8.21 ± 0.333	7.93 ± 0.344	7.43 ± 0.463	7.11 ± 0.529
NIR Index	32.37 ± 19.10	37.14 ± 17.62	30.42 ± 17.95	27.66 ± 21.34
TWI Index	47.87 ± 11.50	45.00 ± 8.08	39.71 ± 12.25	37.16 ± 9.06
TLI Index	67.50 ± 19.51	68.85 ± 17.05	65.71 ± 12.72	55.50 ± 25.68

*Note:* Results of the controls conducted at different time points (1st to 4th control) with mean values ± standard deviation for the variables of pain, pH, NIR index, TWI index, and TLI index. Pain values show a progressive decrease over the controls, while pH also gradually decreases. The NIR, TWI, and TLI indices fluctuate across the different time points.

Abbreviations: NIR, Near Infrared Reflectance Index; TLI, Tissue Luminance Index; TWI: Tissue Water Index.

The correlation between each of these variables and the wound area was studied, and no statistically significant correlation was found (*p* > 0.05).

**FIGURE 3:**
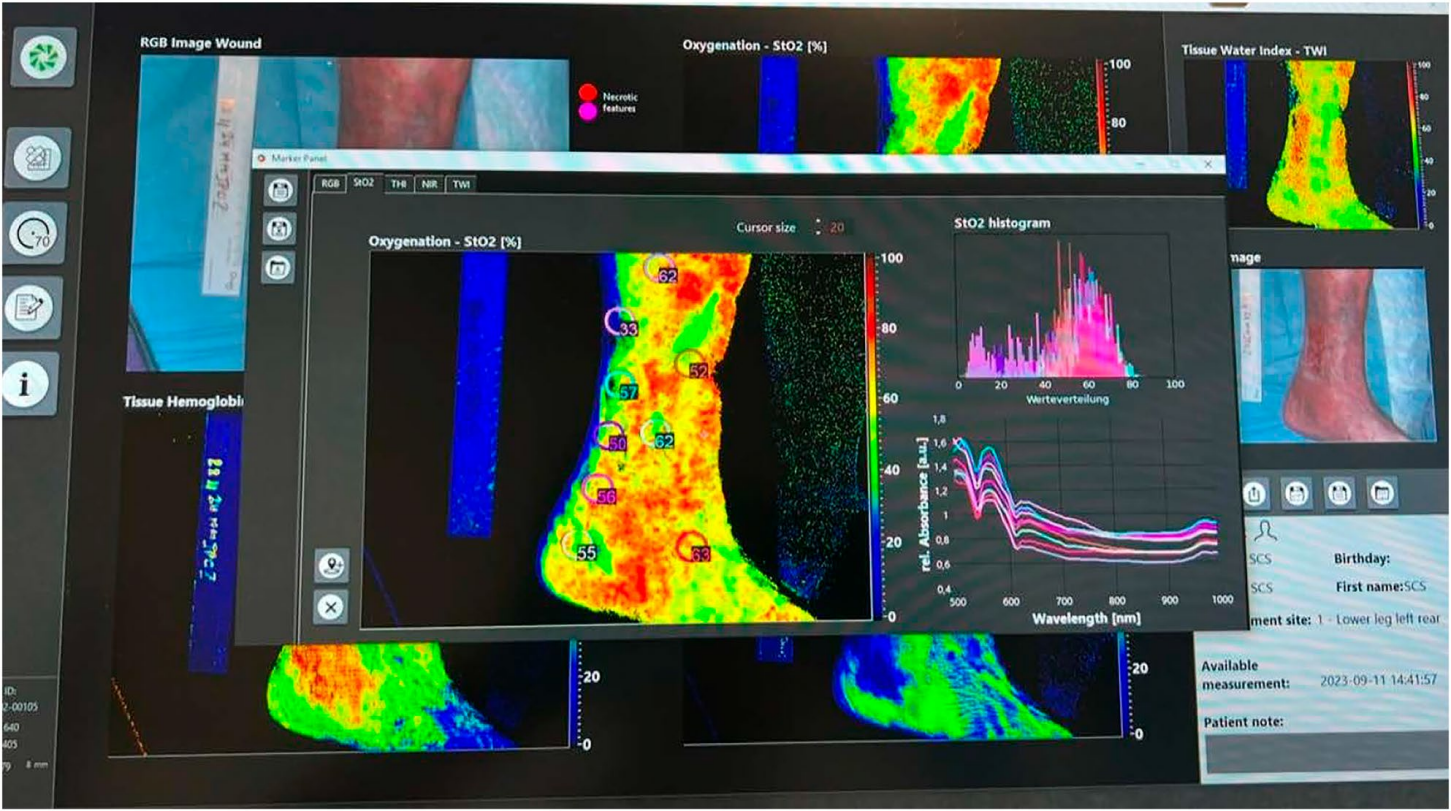
Image of the software used for the quantification of temperature, tissue oxygenation, NIR, TWI, and TLI. The TIVITA hyperspectral imaging system was employed to analyze the variables of the study. *Source*: Own data.

The correlation between wound size, pH, Sat. O_2_, and temperature (Tª) was studied. No statistically significant correlation was established with the temperature variable, although a significant correlation was found between Sat. O_2_ and pH (*p* < 0.005), as measured by Spearman's Rho. Figure 3 displays the software used for the quantification of temperature, tissue oxygenation, near‐infrared imaging, tissue water index, and tissue lipid index.

### 3.1 Quality of Life Assessment

Regarding the evaluation of quality of life in people with wounds, the Quality of Life Today, EQ‐5D‐5L, and QoL‐17 scales were used. The data is shown in Table 3.

**TABLE 3:** Evolution of Quality‐of‐Life Scales.

	EQUATION 5D‐5L ‐pre	EQUATION 5D‐5L ‐post	Wound QoL 17 ‐pre	Wound QoL 17 ‐post	Quality of life today ‐pre	Quality of life today ‐post
Mean	0.516	0.841	1.752	0.532	68.13	90
Median	0.527	0.841	1.700	0.580	70	85
Standard deviation	0.179	0.101	0.453	0.243	6.455	8.839
Percentiles 25						
50	0.366	0.818	1.305	0.290	60	85
75	0.527	0.841	1.700	0.580	70	85
	0.729	0.893	2.200	0.700	75	95

*Note:* Summary statistics for the quality‐of‐life measures at different time points. The table shows the mean, median, standard deviation, and percentiles (25th, 50th, and 75th) for the EQUATION 5D‐5L scores, Wound QoL 17 scores, and Quality of Life Today scores. The EQUATION 5D‐5L and Wound QoL indices show a general improvement over time, with higher scores indicating better quality of life. The standard deviation reflects the variability of responses within each group.

The mean differences for paired samples were compared, and statistical significance was observed with a significance level of *p* < 0.01.

## 4 Discussion

Considering the objectives of this study, it is important to highlight that this study is based on preliminary data obtained from a small sample of patients, which limits the generalisability of the results. One of the results obtained was a reduction in the wound area by 10 cm^2^, which was statistically significant. Four lesions had completely healed before the end of the observation period. These results are similar to those found in the study by Helena et al. [22].

Regarding the evolution of the pH, it shifted from alkaline to a neutral pH, indicating favourable progress. Considering that chronic wounds are typically alkaline, a decrease in pH and a trend toward acidification can be seen as indicators of a good prognosis [23]. Acidification, on the other hand, increases the pO_2_ in wounds (Bohr effect). In a previous study, Leveen et al. [24] determined that a pH drop of 0.9 units multiplies oxygen release by five. Therefore, lowering the pH also improves superficial oxygenation of the lesion.

As previously mentioned, the hyperspectral lamp is capable of measuring the chromophores of haemoglobin and its derivatives (oxyhemoglobin, deoxyhemoglobin, and water), providing a map of skin and subcutaneous oxygenation (Sat.O_2_ and the NIR index) and the relative haemoglobin index in the tissue. In the study by Grambow et al. [8], the effectiveness of the hyperspectral lamp for evaluating Peripheral Arterial Disease (PAD) was assessed by measuring Sat.O_2_ and the NIR index. In our study, the Sat.O_2_ index increased from 57% to 61.3%, indicating a 4‐point rise in the oxygenation of the wound bed, a result similar to what was indicated by Leveen et al. [24]. However, this was not the case for the NIR index or perfusion index, which increased during the first observation, as angiogenesis was expected to be occurring, improving perfusion. In subsequent observations, the NIR index decreased, even though the Sat.O_2_ continued to rise. One way to understand this process could be that the angiogenesis phase has already been surpassed, and the higher concentration of oxygen is transferred to the superficial layers, where granulation is taking place and where the most oxygen is required, at the expense of deeper tissue. This is a finding we cannot objectively validate as we lack the means to do so.

Through spectral light and colorimetry, we can observe subcutaneous water (TWI), providing extra information about the cellular process. Wounds are associated with the presence of edema, or in other words, an increase in the filtration of liquid from the intravascular to the interstitial space, which is related to inflammation or obstruction of the lymphatic system. Therefore, evaluating the water level in the wound environment gives us an idea of how inflammatory processes are resolving as this excess water decreases [25]. In the study by Stamatas et al. [26], it was concluded that spectral imaging was a valuable, non‐invasive tool in studying edema pathology and could be used to monitor its evolution. In our study, the tissue water index (TWI) decreased by nine points, indicating a reduction in edema in the wound area. Therefore, we can say that the dressing under study helps reduce edema and, consequently, inflammation. This corroborates the action of the high‐capillarity dressing, which is known to exert a negative pressure of 68 mmHg.

During the four clinical observations, a notable reduction in superficial and deep infection indicators was observed. Initially, four patients presented clinical signs of infection, but by the end of the process, none showed such signs. It is important to note that these patients did not receive antibiotic treatment throughout the process, suggesting that the dressing used, likely due to its ability to effectively absorb excess exudate, also contributed to reducing bacterial load.

In this study, the use of a calcium alginate dressing combined with the high‐capillarity dressing was chosen based on their complementary properties. The calcium alginate dressing was selected for its high absorbent capacity, which is especially beneficial for wounds with high exudate levels [27]. While the high‐capillarity dressing was chosen for its ability to maintain a moist wound environment, facilitate capillary action, and promote healing, the calcium alginate dressing was incorporated to optimise fluid management in wounds with significant exudate. Alginate's ability to effectively absorb exudate and form a gel‐like consistency helps maintain an optimal moisture balance, which is crucial for preventing maceration and promoting tissue granulation. The combined use of both dressings was designed to maximise healing potential, particularly in more challenging wound types where fluid control is a key factor in the healing process. This finding highlights the potential of the dressing not only in controlling exudate but also in modulating the microbial environment of the wound.

One of the limitations of this study is the small number of participants, which may have influenced the generalisability of the results. Although this pilot study provides valuable insights into the effectiveness of the high‐capillarity dressing, it is acknowledged that a larger sample size would be necessary to confirm these findings and assess their applicability in a broader population. This limitation highlights the need for further research with larger samples to validate the results obtained.

## 5 Conclusion

The results obtained in this pilot study suggest that the high‐capillarity dressing improves chronic wound healing by reducing the wound area, promoting pH acidification, and improving superficial oxygenation. It also reduces edema and eliminates signs of infection without the need for antibiotics, suggesting effective control of exudate and the microbial environment. These findings support its efficacy in the comprehensive management of chronic wounds and highlight the need for further research into its clinical application.

## Disclosure

This study complies with the established principles of ethics and integrity to ensure transparency and rigour in research.

## Ethics Statement

The study was approved by the Research Ethics Committee of the University of León under internal registration number ETICA‐ULE‐053‐2024.

## Conflicts of Interest

The authors declare no conflicts of interest.

## Data Availability

The authors have nothing to report.
